# Progress and challenges for understanding the function of cortical microcircuits in auditory processing

**DOI:** 10.1038/s41467-017-01755-2

**Published:** 2017-12-18

**Authors:** Jennifer M. Blackwell, Maria N. Geffen

**Affiliations:** Department of Otorhinolaryngology: HNS, Department of Neuroscience, Neuroscience Graduate Group, Computational Neuroscience Initiative, University of Pennsylvania, Philadelphia, PA 19104 USA

## Abstract

An important outstanding question in auditory neuroscience is to identify the mechanisms by which specific motifs within inter-connected neural circuits affect auditory processing and, ultimately, behavior. In the auditory cortex, a combination of large-scale electrophysiological recordings and concurrent optogenetic manipulations are improving our understanding of the role of inhibitory–excitatory interactions. At the same time, computational approaches have grown to incorporate diverse neuronal types and connectivity patterns. However, we are still far from understanding how cortical microcircuits encode and transmit information about complex acoustic scenes. In this review, we focus on recent results identifying the special function of different cortical neurons in the auditory cortex and discuss a computational framework for future work that incorporates ideas from network science and network dynamics toward the coding of complex auditory scenes.

## Abstract (web summary)

Advances in multi-neuron recordings and optogenetic manipulation have resulted in an interrogation of the function of specific cortical cell types in auditory cortex during sound processing. Here, the authors review this literature and discuss the merits of integrating computational approaches from dynamic network science.

## Introduction

**Fig. 1:**
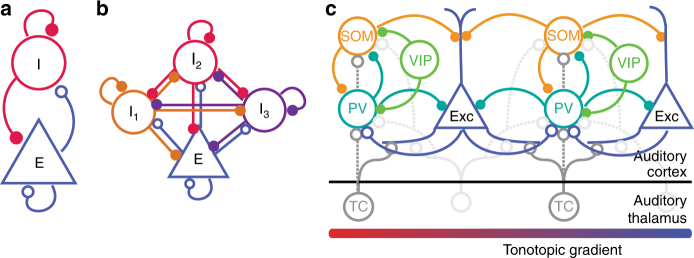
Simplified views of cortical circuits. (**a**) Diagram of excitatory–inhibitory circuit with recurrent connections. Theoretical and experimental studies demonstrate that inhibition stabilizes between excitatory and inhibitory neurons in the auditory cortex. (**b**) Inhibitory–excitatory network can be extended to include several interneuron subtypes. (**c**) Schematic diagram of connectivity between select neurons in the auditory cortex (note that layer-specific information is omitted here): Exc: Excitatory neurons; PV: parvalbumin-positive interneurons; SOM: somatostatin-positive interneurons; VIP: vasopressin-positive interneurons; TC: Thalamo-cortical projection neurons. All neuron types receive additional inputs from other brain areas, which were omitted from the diagram for simplicity. Open circles: excitatory synapses; closed circles: inhibitory synapses. Solid lines indicate dominant projections; dashed lines indicate occasional connections

In auditory processing, a long-standing question has been the function of cortical architecture in specific sensory functions. Auditory cortex (AC) is comprised of neurons of many different types, providing the ability to perform an astonishing number of computations. Even the most basic distinction between neurons into excitatory and inhibitory units markedly expands the computational capacity of a network, and a quest in auditory neuroscience has been to unravel the function of specific microcircuits in sound encoding and plasticity. A particularly interesting aspect of cortical connectivity is the diversity of inhibitory neurons in their morphology and synaptic properties^1^ (Fig. 1a). Interneurons form reciprocal connections not only with the excitatory neurons, but also with each other (Fig. 1b). Furthermore, the diversity of connections between different types of inhibitory interneurons can affect how information is processed in the network (Fig. 1c).

Recent advances in optogenetics and imaging^2^ of the role of cortical circuits comprised of distinct inhibitory neurons in basic auditory functions, such as frequency discrimination and adaptation to temporal stimulus statistics. In combination with computational techniques for inhibitory–excitatory network analysis, these experimental approaches offer promise for unraveling the microcircuits within AC for representing sounds. Here, we discuss progress and limitations in our understanding that emerges from recent investigations of the function of cortical microcircuits in audition.

## Role of inhibition in auditory frequency discrimination

Spectral differences between sounds are fundamental cues for identifying a dangerous sound, be it the sound of an approaching predator or screeching brakes; recognizing a familiar speaker; or distinguishing different animal vocalizations^3–5^. Frequency selectivity, originating with spectral decomposition of the acoustic signal by the cochlea, is a strong organizing feature of neuronal responses in the auditory pathway. Neurons in the AC exhibit frequency selectivity^6–8^, responding to a subset of frequencies more strongly than others. This selectivity is thought to support perceptual frequency discrimination acuity^9–11^ (but see refs. ^12,13^): the greater the difference either in individual or population neuronal responses for tones of neighboring frequencies, the higher frequency discrimination acuity. Either more narrowly tuned neurons, or neurons with higher signal-to-noise ratio in tone-evoked responses would support higher frequency discrimination acuity, as the difference in responses to neighboring tones will be higher in these neurons than in broadly tuned/low signal-to-noise neurons. One of the most extensively tested roles of cortical inhibition in auditory processing has been in shaping frequency selectivity of excitatory neurons. Inhibition may sharpen frequency tuning and increase the signal-to-noise ratio in excitatory tone-evoked responses by suppressing spontaneous excitatory activity; alternatively, either broad or co-tuned inhibitory inputs may sharpen frequency selectivity owing to the rectifying non-linear integration (such as the spiking non-linearity)^14–16^. Differential timing of excitatory and inhibitory co-tuned inputs can further refine frequency tuning of excitatory neurons^17^. Experimental evidence from pharmacological experiments and intracellular recordings has supported either effect^8,14,17–20^. An interesting possibility is that distinct inhibitory neuronal cell types may contribute differentially to shaping frequency selectivity. The development of optogenetic manipulations has promised to disambiguate the effects of different specific neuronal cell types^21^.

**Fig. 2:**
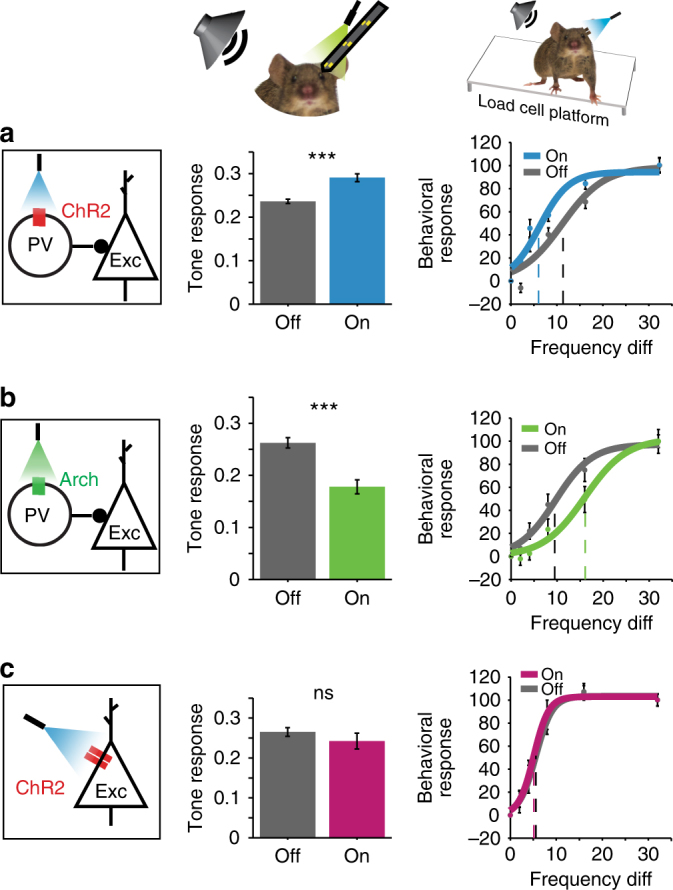
Inhibitory interneurons affect auditory cortical responses and behavior. Activating PVs with ChR2 (**a**) increases tone-evoked responses and improves behavioral frequency discrimination acuity, whereas suppressing PVs using Arch has the opposite effect (**b**). (**c**) Direct activation of excitatory neurons with ChR2 does not change tone-evoked responses or behavioral frequency discrimination acuity on average. (**c** **e**) Left: diagram of optogenetic manipulation. Center: mean tone-evoked response magnitude under light-off and light-on conditions based on neuronal recordings. Right: Behavioral response to a shift in frequency under light-off and light-on conditions. Adapted from ref. ^23^

A series of studies that optogenetically manipulated the most common interneuron type, parvalbumin-positive interneurons (PVs) confirmed that inhibitory neurons modulate frequency tuning in AC: activating PVs enhanced feedforward connectivity between excitatory units. The spontaneous activity of excitatory neurons was decreased, and the frequency tuning width was narrower, increasing frequency selectivity^22^. Consistently, PV activation also increased the strength of tone-evoked responses and improved behavioral frequency discrimination acuity, whereas suppression decreased the strength and tuning width of the tone-evoked responses in putative excitatory neurons, and drove an impairment in behavioral frequency discrimination acuity (Fig. 2a–c)^23^.

But are the effects different between distinct interneuron types? There is a range of interneuron classifications available, with at least two major groups of neurons emerging besides PVs, somatostatin (SOMs), and serotonin receptor 5HT3aR, of which a major group are also positive for vassopressin (VIPs)^1,24,25^. Although each interneuron class includes a number of different cell types and may change with development and experience^26^, these classes of neurons have received prominent attention, as they approximate a canonical cortical circuit (Fig. 1), and owing to availability of transgenic mouse lines. Optogenetic activation of either PVs or SOMs exerted a similar mix of effects on tone-evoked activity in excitatory cells, with activation providing either multiplicative scaling, as would be expected from co-tuned inhibition, or linear amplification, as would be expected from broad inhibitory inputs^27^. This variability in combination with spiking threshold non-linearity and strength of suppression across different neurons can both amplify and sharpen tuning properties of excitatory neurons^27^. On average, suppressing interneurons had differential effects: suppressing SOMs increased the gain of excitatory neurons, whereas suppressing PVs weakened frequency tuning^28^. Nonetheless, as with activation, in individual neurons inactivation of either type of interneuron showed a range of effects, thus supporting a number of models for interactions between PVs, SOMs and excitatory neurons. Measuring the tuning widths of individual PVs, excitatory neurons and SOMs neurons furthermore did not yield clear distinctions^29,30^, potentially because these classes of neurons are themselves comprised of multiple cell types. These differences may be exacerbated across studies by the various biases toward specific subclasses by different recording techniques. Indeed, a recent review of SOMs estimated over 100 subtypes of SOM neurons^31^. Thus, whereas the optogenetic perturbations of PVs and SOMs confirmed their role in shaping frequency tuning in the AC, the results were consistent with the range of results in the pharmacological literature, and a clear distinction between the function of the two interneuron subtypes in frequency tuning has not emerged.

By targeting the electrophysiological recordings to specific cell types, it has begun possible to assess the diversity of neuronal responses in both stimulus selectivity and time course. Interestingly, in the temporal domain, responses of PVs had faster onsets on average than excitatory neurons, whereas SOM neurons exhibited slower response onsets than excitatory neurons^29,30^. Such differential activation timing may provide an additional mechanism for sharpening of tone-evoked responses in AC^32^: the differences between inhibitory and excitatory response onsets would drive more precise response onsets over the excitatory population. The timing differences in suppression between PVs and SOMs could support a number of other functional effects, for example when applied to suppression of responses owing to the stimulus history. These observations, facilitated by targeted recordings, provide information about specific neuronal synaptic parameters that should inform the design of computational models. Future studies are required for understanding whether the timing differences in tone-evoked responses of PVs and SOMs results in distinct function of these neurons in frequency discrimination.

## Role of inhibition in adaptation to stimulus statistics

**Fig. 3:**
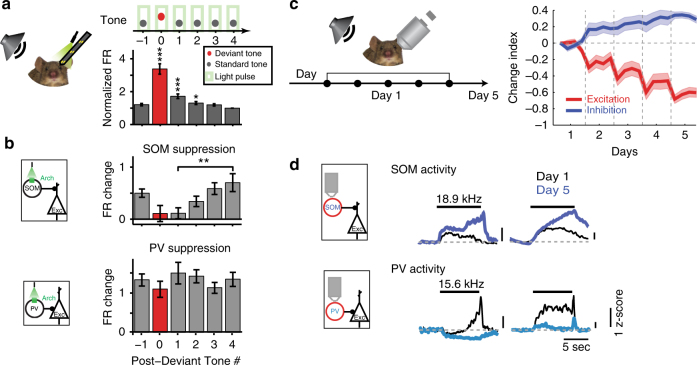
Specific inhibitory neuron type mediates auditory adaptation. (**a**) Top: The effect of SOM and PV inactivation on stimulus-specific adaptation to frequent tones was tested using an oddball stimulus, with two tones at 10–90 ratio, light every 5th tone. Bottom: The mean firing rate (FR) during repeated tones adapted with successive presentations of the standard tone. (**b**) SOMs provide stimulus-specific inhibition, as the effect of SOM suppression increased with repeated standard tones. PVs provided constant inhibition regardless of adaptation. **a**, **b** adapted from ref. ^34^. (**c**) Passive exposure to a tone stimulus lead to a decrease in excitatory and an increase in inhibitory activity over 5 days. Left: calcium activity was imaged using two-photon microscopy in populations of identified inhibitory and excitatory neurons before and after subjecting the mouse to prolonged exposure to tones. Neuronal activity was measured as spike counts inferred from the imaged fluorescence signal. Right: change index of the mean activity in response to the tone to which the mouse was exposed, averaged over populations of excitatory (red) or inhibitory (blue) neurons, over days since prolonged tone exposure onset. Mean excitatory activity decreased with exposure, whereas mean inhibitory activity increased. (**d**) Among the inhibitory neurons, the activity of SOMs increased following passive tone exposure, whereas the activity of PVs decreased. Mean z-scored time course of Calcium activity of SOMs or PVs in response to a tone at day 1 (black traces) and day 5 (*blue* traces). **c**, **d** adapted from ref. ^37^

Time processing is particularly important for auditory perception. Perception is formed as much by the present stimulus as by the history of preceding stimuli; it is an interaction between the representation of new events and memory of past events. Sensory cortex constantly reshapes responses to present stimuli dependent on the context. Responses of nearly all neurons in the AC (95%) change with stimulus temporal history, exhibiting stimulus-specific adaptation (SSA)^33,34^. Typically, the firing rate is reduced in response to repeated stimuli through a process that involves integration of stimulus statistics over time^33,34^. Inhibitory–excitatory networks support this transformation, and PVs and SOMs were found to play a differential role in cortical adaptation. SSA is quantified as the index of change in the firing rate in response to a rare ‘deviant’ tone presented as part of a sequence with another common ‘standard’ tone, at varying presentation probabilities (for example, 10%, deviant vs. 90% standard). SSA is thought to be supported by a combination of thalamo-cortical depression and intra-cortical inhibitory–excitatory circuit effects^35^. Indeed, suppressing either PVs or SOMs optogenetically increased the strength of adaptation in excitatory neurons^34^ (Fig. 3a, b). However, PVs and SOMs differed in their contribution to adaptation: suppression of SOMs evoked a stimulus-selective increase in excitatory responses to the standard, but not the deviant tones, whereas suppressing PVs led to a non-specific response increase. When examining the effect of SOM suppression on excitatory responses to repeated presentations of the standard tones following a deviant tone, disinhibition of the excitatory response increased with repeated presentations (Fig. 2a–c), further confirming that SOMs provided a selective inhibitory contribution to SSA. PV and SOM interneurons themselves both exhibit SSA^34,36^, so that the unique contribution of SOMs in stimulus-specific inhibition of excitatory responses was possible through selective suppression of this cell type.

Further work revealed that stimulus-specific inhibition mediated by SOMs persisted over a longer time scale, in habituation following passive exposure to sounds over several days^37^ (Fig. 3c, d). Whereas the excitatory and PV neuronal responses to habituated sounds were reduced over several weeks of exposure, SOM responses increased, as did inhibition from SOMs. Thus, in temporal domain, the function of SOMs is consistent with regulation of the gain of cortical responses to sounds based on their behavioral prominence or relevance. SOMs thus contribute to adaptation and habituation, acting on several time scales to control the gain in response to commonly presented acoustic stimuli, exerting a more specific modulation than PVs. Such modulation of excitatory activity may contribute to the more general context-specific gain modulation and adaptation observed within AC^38^. Indeed, SOMs similarly play a specialized role in driving more general adaptation to temporally repeated tones^39^, and exert a differential effect on excitatory neuronal responses than PVs depending on the preceding stimulus^40^. Thus, the temporal history of the stimulus is important for the differential function of interneuron modulation. This functional dissociation likely underlies other temporally differentiated functions, such as integration of stimulus sequences, or more general computation of spectro-temporal statistical regularities in sound sequences. A promising direction for future studies would be to continue the exploration of the function not just over the instantaneous responses to tones, but in understanding how, over a range of time scales, inhibition may modulate dynamic changes in sound response properties.

## Inhibitory cascades within the AC

Although the above studies mainly considered the effects of PVs and SOMs on excitatory neurons, understanding how PVs and SOMs act within a cortical microcircuit is of particular importance given the modulatory roles these interneurons play when targeted by feedback from other brain regions and different neuromodulatory projections. In a circuit that supports reduction of auditory responses during locomotion, a subset of secondary motor cortex neurons, which are active during movement, suppresses excitatory tone-evoked responses in AC by activating PVs, which in turn suppress excitatory neurons^41,42^. In addition, PVs are involved in inter-hemispheric information integration, as callossal projections terminate on PVs, which suppress cortico-cortical excitatory neurons^43^. PVs and SOMs can be activated by oxytocin, which likely supports the sharpening of responses to pup calls observed in mothers^44^. PVs also shape cortical responses to tones coupled with aversive stimuli^45^, as part of circuit, which includes projections from the amygdala to layer-1 neurons in AC. Thus, projections from cognitive and emotional brain centers likely preferentially target inhibitory interneurons, and may affect behavioral and emotional processing.

Other inhibitory neuronal subtypes further contribute to inhibitory cascades in the cortex. For example, there is extensive evidence that PVs and SOMs are regulated by VIPs. Cortical VIP interneurons are recruited by projections from other brain regions^46,47^ and can be modulated by cholinergic projections^48–51^ allowing for external control of these local microcircuits. In the AC, engagement in an auditory task enhanced the activity of PVs, SOMs, and VIPs, attributed to cholinergic modulation^51^. Activation of VIP interneurons disinhibited excitatory responses^52^, consistent with an additional inhibitory synapse between VIPs, another inhibitory neuron and excitatory neuron. Indeed, in vitro activation of VIPs suppresses PV and SOM activity, thereby providing a mechanism for delayed activation of excitatory tone-evoked responses. Interestingly, VIP neurons were driven by sounds at much lower intensities than either PV or SOM neurons^53^. The increased excitatory neuronal activity due to VIP activation may contribute to increased gain of sensory inputs, although it remains to be determined whether the relative timing of disinhibition may provide an increase in gain, rather than non-specific elevation in cortical activity. VIP neurons have also been implicated in integration of cross-modal activity, as responses of VIPs in the visual cortex are suppressed by sound^54^. This wide range of effects of VIP activation suggests that the connections between VIPs and inhibitory and excitatory neurons are likely modulated in a task- and modality-specific fashion, and therefore the interpretation of their function should inherently be studied in a specific statistical and behavioral context. To understand whether and how these circuits integrate with each other and what biophysical constraints are required for their function, the results of these studies need to be incorporated in a circuit-level model that include interactions between the different circuits.

## Caveats in optogenetic result interpretation

In interpreting these rich and varied experimental results, it is important to account for limitations and potentially confounding factors: An important caveat to grouping the interneurons into classes based on molecular markers is that each of these classes are comprised of multiple interneuron subtypes, and those subtypes are distributed differentially across the different cortical laminae. For instance, PV interneurons are comprised of not only two already diverse large groups of neurons, basket and chandelier cells, but also of a number of other neurons^1^. SOM interneurons include Martinotti cells, whose axons target the distal dendrites of pyramidal neurons, as well as at least two other classes of layer 2/3 targeting neurons^55^. A recent review estimated between 4 and 100 subtypes of SOMs depending on classification method, such as differential labeling and projection patterns^31^. Incorporating some specific aspects of neuronal morphology, such as by building multi-compartment neuronal models and accounting for expression of different molecules involved in neuronal communication, may prove essential for differentiating between the effects of dendrite-targeting SOMs and cell body targeting interneurons. Indeed, the excitatory–inhibitory circuit composition likely differs between cortical layers with some neuronal types being overrepresented and targeting different parts of the excitatory cell body, leading to differences in integration and non-linearity^56–58^. Different recording techniques, such as extracellular recordings of activity of optically tagged neurons vs. two-photon guided patch-clamp recordings might be biased toward different subclasses within the optogenetically identified groups and different cortical sublayers, potentially reporting conflicting results on the response properties of different cell types^29,30^. By measuring a number of essential connectivity parameters, the strength and time constant of synapses between different neuronal types within different layers, it should be possible to further develop models to extrapolate the results across different cortical connectivity patterns. In turn, electrophysiological experiments, including multiple-neuron intracellular recordings, could be used to establish the specific parameters for connectivity between different neuronal cell types^59^. The computational approaches to implement such detailed biophysical models have been developed, and, as detailed in the next section, a small number of studies started using these computational frameworks to build models incorporating multiple neuronal subtypes in a variety of circuit motifs.

## Computational models for excitatory–inhibitory interactions

**Fig. 4:**
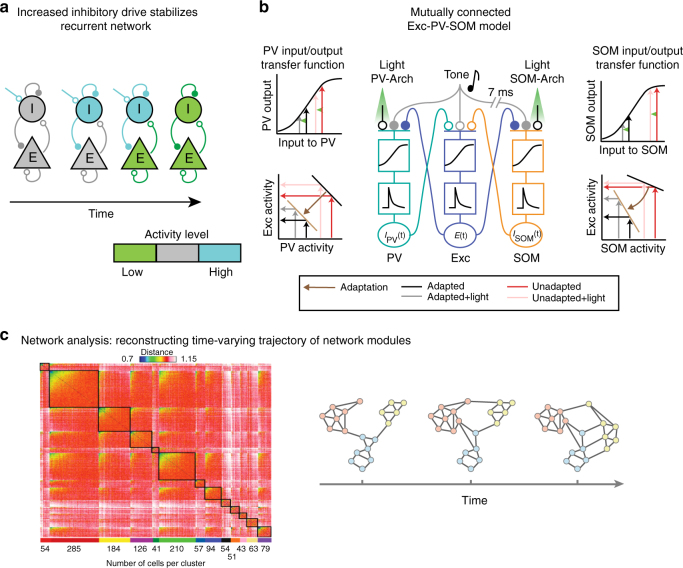
Progressively complex view of cortical dynamics. (**a**) Diagram of the time course of inhibition-stabilized recurrent dynamics. Adapted from ref. ^62^. (**b**) Reduced model of mutually coupled Excitatory (Exc)—PV—SOM network. Exc rate is a non-linear—linear function of excitatory synaptic inputs (filled circles) evoked by the tone, as well as inhibitory inputs (open circles) from PVs and SOMs. PV and SOM firing rate is a non-linear—linear function of excitatory synaptic inputs from excitatory cells and tone-evoked excitatory inputs. Optogenetic manipulation by Arch is modeled as an inhibitory synaptic input. Adapted from ref. ^34^. (**c**) Left: network modules identified based on the correlation network structure. Adapted from ref. ^95^. Right: diagram of the time course of transformation in brain network structure with learning: Nodes belonging to the same module are colored in the same color. Black lines refer to the edges of the network. Note that with learning, the connectivity within and between modules is transformed. Adapted from ref. ^101^

The approaches for incorporating inhibition into modeling sensory cortical function have ranged from implementing massive inhibition, forming inhibition-stabilized networks^60–62^ to fine-tuning parameters of individual neuronal feedback circuits^63^. Indeed, the large range of response profiles to activation of interneurons in inter-connected networks was extensively studied in the context of inhibition-stabilized networks that interpret the function of inhibition as supporting “stability” in neuronal pattern discharges across neuronal population activity^62,64,65^. Stabilization of excitatory neuron activity by recurrent inhibition can be explained by analyzing the dynamics of firing rates of excitatory and inhibitory populations over time, including feedback propagation of activity (Fig. 4a). This relatively simple model also explained heterogeneous findings from experiments testing the effect of increasing inhibition on network activity^23,27,28^ as providing input to inhibitory neurons resulting in increased activity of excitatory neurons in a model of an excitatory–inhibitory network^60^. Additional studies of inhibition-stabilized circuits focused on the role of inhibition in improving tuning of neurons for specific sensory features, such as orientation selectivity in the visual cortex using more abstract supralinear networks with feedback inhibition^65^, and the ability to tightly track the stimulus fluctuations in a balanced excitatory–inhibitory regime^32^. Extending the inhibitory–excitatory network model to incorporate the connectivity patterns between different types of interneurons, including PVs, SOMs, and VIPs, required a different set of constraints for explaining cortical tuning properties than when only one inhibitory subtype was used^66^ (Fig. 1b). Moreover, the resulting network did not require the massive inhibitory feedback consistent with inhibition-stabilized networks to model the observed effects for tuning properties. This was particularly important for understanding the tuning properties of neurons in layers 2/3 of the visual cortex, where only weak inhibition was identified experimentally.

In the AC, simpler models have already proved useful for understanding the heterogeneity of the effects of optogenetic manipulations. A static model illustrated the origin of the heterogeneous effects of optogenetic perturbations of PVs and SOMs on excitatory neuronal activity by shifting the threshold as well as the strength of inhibitory–excitatory conductances^27^. This model demonstrated plausibility that linear scaling and additive effects are produced by the same underlying mechanism—addition followed by rectification. A mutually coupled excitatory–inhibitory firing rate model reproduced the differential effects of manipulating PVs and excitatory neurons on the baseline and tone-evoked responses by adjusting the non-linearity at the inhibitory–excitatory synapse^23^. Differential synaptic strength of connections between the excitatory and inhibitory neurons could account for the differences between SOM and PV effects on adaptation in excitatory neurons, including flat and saturating non-linearities for synapses between PVs or SOMs and excitatory neurons, respectively (Fig. 4b). The principles outlined by the simplified models illustrate that by manipulating a specific aspect of input integration, the same wiring pattern can produce the heterogeneous results observed experimentally.

Several additional studies have used similar approaches to explore the role of distinct interneurons in cortical processing across sensory modalities. Using a combination of anatomical and optogenetic data in the somatosensory cortex^67^, a model could identify correlations in connection strengths between different neuronal subtypes^68^. Furthermore, a model of mutually coupled, fast-spiking, and non-fast-spiking interneurons, revealed the limitations in the role of fast-spiking neurons in cortical oscillations^69^. Beyond the sensory cortex, model circuits incorporating several inhibitory interneuron subtypes exhibited recurrent memory across a number of biophysically plausible configurations^70,71^. Indeed, recurrence in neuronal circuits increased the network’s capacity to efficiently store and recall memories^72^, as originally proposed^73^. Measuring whether and how inhibitory neuronal populations control and contribute to recurrent activity using recently developed methods for efficient model training^63^ should prove a fruitful way forward for understanding not only the key cell types that are affected by optogenetic perturbations, but also the time scales of their modulatory effects.

The temporal impact of optogenetic manipulations on auditory activity might differ between opsins, and thus have variable behavioral effects^74^. Expanding current models to account for the effects of inhibition at a circuit level will clarify how inhibition shapes trajectories of neuronal population dynamics^75^. In a complex network, where stimulus-evoked activity and neuronal connectivity patterns are highly heterogeneous^23,76,77^, computational models incorporating mutually coupled excitatory–inhibitory cells can reveal network features that may otherwise be obscured by results of electrophysiological and imaging experiments, which are biased toward stronger connections^78^. Recurrent circuit dynamics may be the dominating feature of cortical circuits, and the interpretation of results based on optogenetic perturbations need to incorporate feedback dynamics in their design^79–81^.

As the number of simultaneously observed neurons has increased with recent advances in functional imaging and dense electrophysiological recording techniques, there is a growing need to efficiently represent how units in a large population relate to one another and how these relations change over time^82^. To meet these demands, current computational approaches address the dynamics of neuronal populations that exhibit non-random dynamics and form higher-degree connectivity^59,83,84^. The study of the structure of excitatory–inhibitory connectivity can be combined with synaptic organizational principles to understand the basis for cortical dynamics^80,85^.

One such approach is to apply principles from network science to tracking the dynamics of neuronal cortical populations (Fig. 4c). Network science has been extensively used to characterize large-scale brain networks, revealing modular, hierarchical or random organization^86,87^. There is extensive evidence that neuronal populations exhibit stereotyped, temporally precise tone-activated patterns of activity in AC^88^, which are repeated during spontaneous firing, reflecting stereotypical population activity organization^89^. Such population activity patterns may differ between the synchronized and the desynchronized state of the cortical network^90^, and identifying inter-connected modules using network science methods can reveal whether and how the modules are transformed between the different brain states. Shared variability in neuronal populations can potentially be explained by distinct patterns of connections between neurons^91^, whereas diverse response patterns may correspond to different coupling patterns between single neurons and neuronal populations^92^—network analysis of activity in cortical slices already identifies functional modules, that exhibit similar organization across sensory cortex^93,94^ (Fig. 4c). Two-photon imaging of calcium activity in large networks of neurons identified similar population activity modes in the AC^95^. Such modes correlated with behavioral responses^96^. Analyzing neuronal activity in terms of population firing rate or activity variability discounts the complex temporal structure of these cellular networks, and thus might underestimate the information contained in neuronal responses^90,97^. Network analysis can furthermore reveal whether and how the neurons within functional modules reorganize with adaptation and learning, and whether specific inhibitory neurons assume specialized roles within networks.

## Outlook/future directions

The diversity of these computational approaches for modeling the function of excitatory–inhibitory circuits and population neuronal activity in cortical sensory processing provides for the basic framework for moving forward in identifying the cortical circuits involved in auditory scene analysis. The complex effects of network interactions can only partially be understood in the context of natural audition when tested with a limited set of isolated stimuli. To the extent that we can view audition through the lens of temporal processing, recurrent inhibitory–excitatory networks can yield unique capabilities for storing and recalling complex temporal sequences, detecting unexpected events, and recognizing patterns of activation characteristic of more complex stimuli^79–81^, such as speech/vocalizations/music. The models should be fitted on data from experiments that would incorporate progressively complex acoustic stimuli; constructed either by varying spectro-temporal complexity, based on the scale-invariant statistical structure of environmental sounds^98,99^, or using statistical methods for shaping random signals to match different sound textures^100^. Taking advantage of the full computational toolset provided by inhibitory–excitatory network modeling, recurrent network dynamics and network science will allow us to tackle the complex richness of cortical circuits, and generalize results across sensory modalities and behavioral paradigms.

The use of dynamic analysis tools to explore sets of possible neuronal activity regimes makes inhibitory–excitatory networks a powerful framework for testing hypotheses on population responses in the cortex. The expansion of these models to include different cell types and wiring schemes in combination with analysis of the network structure dynamics is required for understanding the functions and sources of variability within specific neuronal populations. To fully understand cortical network function, our computational models must take into account the field’s wealth of data concerning neuronal subtypes, how they are connected locally and to other areas, how they respond to stimuli, and how optogenetic manipulation perturbs them in order to make testable predictions about how these networks behave. Thus, modeling studies need to be combined with a range of experimental techniques that would allow measurement of the strength of synaptic connections between neurons within specific layers and more precisely defined cellular classes.
